# Patient Perspectives on Prostate Cancer Prehabilitation: A Qualitative Study Using the Behaviour Change Wheel to Investigate Barriers and Facilitators to Engagement

**DOI:** 10.3390/curroncol33090564

**Published:** 2026-09-17

**Authors:** Hannah Harsanyi, Nicole Slot, Ashley J. Housten, Yun Yun Lee, M. Eric Hyndman, Lin Yang

**Affiliations:** 1 Cancer Epidemiology and Prevention Research, Cancer Care Alberta, Calgary, AB T2N 5G2, Canada; hannah.harsanyi@cancercarealberta.ca (H.H.);; 2 Division of Public Health Sciences, Department of Surgery, Washington University School of Medicine in St. Louis, St. Louis, MO 63110, USA; 3 Department of Oncology, Cumming School of Medicine, University of Calgary, Calgary, AB T2N 1N4, Canada; 4 Department of Surgery, Cumming School of Medicine, University of Calgary, Calgary, AB T2N 1N4, Canada; 5 Southern Alberta Institute of Urology, Calgary, AB T2V 1P9, Canada; 6 Department of Community Health Sciences, Cumming School of Medicine, University of Calgary, Calgary, AB T2N 1N4, Canada

**Keywords:** prehabilitation, treatment preparation, prostate cancer, behaviour change, intervention development, qualitative research

## Abstract

Cancer prehabilitation aims to improve outcomes by helping patients physically and mentally prepare for treatment. This qualitative study aimed to investigate barriers and facilitators to engaging in prostate cancer prehabilitation and create recommendations for designing interventions. Semi-structured interviews were used to explore perspectives on cancer prehabilitation among men diagnosed with prostate cancer in Alberta, Canada. Interviews were thematically analyzed to identify barriers and facilitators to engagement. Themes were mapped to the Behaviour Change Wheel (BCW) to create recommendations for intervention development. Thirty-four men diagnosed with prostate cancer completed interviews. Key facilitators to engaging in prehabilitation included opportunities for social connection, strategies to reduce treatment-related side effects, and support coping with the psychological impact of diagnosis. Barriers included competing care priorities, lack of familiarity with prehabilitation, logistical challenges, and stigmatized perceptions of help-seeking. To promote improved patient engagement, prehabilitation interventions should be initiated near diagnosis, offer personalized, goal-oriented interventions with multiple components, and have options for virtual participation to increase accessibility. These recommendations may help improve the uptake and impact of prehabilitation programming in prostate cancer care. Further research to identify evidence-based interventions for mitigating treatment-related side effects may help increase patient motivation to engage in cancer prehabilitation.

## Simple Summary

Prehabilitation uses the time between diagnosis and treatment to help patients prepare for cancer care. To make helpful programs, it’s important to understand what might prevent or encourage patients to get involved. In this study, we had discussions with 34 men with prostate cancer to learn about their experiences and what they viewed as important in the time before treatment. A tool called the Behaviour Change Wheel helped us organize and understand these conversations. We found eleven main themes. Patients expressed that prehabilitation should address not only physical health, but also information needs, emotional support, and practical concerns. Many men felt anxious and uncertain after diagnosis and thought connecting with other patients could help. Because patients were motivated to prevent treatment side effects, more evidence on the benefits of prehabilitation would be useful. This study shows how patient perspectives can be considered alongside clinical evidence to plan prehabilitation programming.

## 1. Introduction

Cancer prehabilitation is a proactive approach that aims to support and prepare patients before they begin cancer treatment, with the aim of addressing current and future deficits that may be associated with their diagnosis and care [1]. Prehabilitation may include interventions such as exercise, nutritional optimization, education or cognitive training, and psychosocial support, or a combination of multiple components [2,3]. Prehabilitation has traditionally focused on preparing patients for the physiological stress of surgery [4] and most research on prehabilitation has been limited to surgical patients [5]. However, cancer treatment extends beyond surgery and there is a growing interest in developing prehabilitation programs to support patients diagnosed with cancer, regardless of the type of treatment they receive [6,7,8,9,10].

For patients diagnosed with localized prostate cancer, active surveillance, surgery, and radiation therapy have been shown to lead to similar survival outcomes [11], meaning that patients have several treatment options for managing their disease [12]. Both surgery and non-surgical treatment options can be physically and emotionally demanding and result in acute, short-term or long-term side effects [13]. Common treatment-related side effects include sexual dysfunction, urinary incontinence, bowel dysfunction, and fatigue, which can significantly impair patients’ quality of life [14,15,16]. In addition, growing utilization of active surveillance may extend the time from diagnosis to treatment in which prehabilitation can be delivered [17,18]. As one of the most prevalent cancer types and among the most well-studied in surgical prehabilitation [5], prostate cancer provides an ideal setting for understanding how interventions can be designed for and delivered to patients across different treatment pathways.

Prehabilitation interventions for prostate cancer patients, such as exercise, pelvic floor training, and stress management, have demonstrated promising results for improving physical function and quality of life [19,20,21,22,23], with limited preliminary evidence for mitigating treatment-related side effects [24,25,26,27]. Although prehabilitation has the potential to improve outcomes for patients who engage with and adhere to interventions, barriers continue to limit uptake and overall effectiveness [28,29,30]. Successful implementation is facilitated by active end-user involvement and consideration of behavioural, contextual, and system-level factors that may influence patient engagement [31,32,33,34]. Furthermore, incorporating patient perspectives into the design of healthcare interventions can help to identify meaningful priorities and create programs with improved relevance, acceptability, and impact [35].

The Behaviour Change Wheel (BCW) offers a theory-based framework to guide the development of behaviour change interventions and policies [36,37]. The core of the BCW is the COM-B model, which outlines capability (C), opportunity (O) and motivation (M) as components that interact to determine behaviour (B). By considering these interrelated sources of behaviour, the BCW provides a guide for developing interventions tailored to the specific barriers and facilitators that influence a target behaviour [37]. Using this framework, our study aimed to characterize patient perspectives on prostate cancer prehabilitation and generate recommendations for the design of interventions that are applicable to patients before prostate cancer treatment.

## 2. Materials and Methods

### 2.1. Study Design

We conducted one-on-one, semi-structured interviews with men who were diagnosed with localized prostate cancer. The study used a qualitative descriptive approach [38] with a hybrid inductive-deductive process. Themes were inductively generated from participant accounts [39] and subsequently mapped deductively onto the Theoretical Domains Framework (TDF) [40], BCW [36], and taxonomy of behaviour change techniques [41]. A qualitative descriptive approach was selected to provide a practice-oriented account of participants’ perspectives and experiences that remained close to their description, consistent with the study aim of identifying practical considerations to inform prehabilitation design. Behaviour change frameworks were used to generate theory-informed recommendations for intervention development, supplemented by specific participant recommendations or endorsements that emerged during thematic analysis. This report followed the Standards for Reporting Qualitative Research (SRQR) guideline (Supplementary File S1) [42].

### 2.2. Participants and Recruitment

Eligible participants were English-speaking adult men diagnosed with localized prostate cancer who had made a treatment decision. Participants were recruited across treatment pathways and were at different stages along the cancer care continuum (from diagnosis to survivorship). Purposive sampling was used to recruit patients in treatment groups categorized as (1) active surveillance (monitoring disease without pursuing immediate treatment), (2) first-line surgical treatment (radical prostatectomy) with or without subsequent lines of therapy, or (3) first-line non-surgical treatment (radiation therapy, brachytherapy, androgen deprivation therapy, and/or cryotherapy). Potential participants were identified from a list of patients receiving care at the Calgary Prostate Cancer Centre who indicated interest in participating in research. Participants were additionally recruited via involvement in a pilot trial of a Tai Chi intervention before radical prostatectomy (NCT05452824); surgical patients who were not involved in prior prehabilitation research were also purposively sampled to ensure interviews were inclusive of patient experiences outside the trial. The study team contacted potential participants to explain the study’s purpose and details of participation. All participants provided informed consent prior to the interview (Supplementary File S2). Sample size was based on data saturation within each treatment group; recruitment within each group ended when coding of two consecutive interviews identified no new themes. Saturation was assessed during weekly researcher meetings to discuss any emerging themes, which were incorporated into the codebook, as appropriate, based on researcher consensus (see Section 2.4).

### 2.3. Data Collection

The interview guide was informed by the TDF [40] and designed to elicit participant responses regarding their personal cancer experience and perceived interest in, purpose of, and outcomes from a cancer prehabilitation program (Supplementary File S3). The interview guide was tested in two mock interviews with researchers external to its development, as well as in two pilot interviews with study participants. Minor revisions to improve wording and flow were made following the pilot interviews. Since the interview guide did not require significant changes, pilot data were included in the analysis.

Virtual or telephone interviews were conducted with participants between August 2023 and March 2024 and were approximately one hour in length. Field notes and reflexive documentation were maintained for each interview. The interviews were audio-recorded and transcribed verbatim. Participants were assigned a unique study identification number and any identifying information was removed from transcripts. In one case, equipment failure resulted in failure to obtain an audio-recording; analysis of this interview was based on detailed notes of participant responses and sentiments written by the interviewer immediately following the interview. In addition to collecting qualitative data, participants were asked to complete a sociodemographic questionnaire. Clinical information on tumor grade was obtained from participants’ electronic medical records of diagnostic biopsy. Participants were offered a $50 CAD gift card to compensate them for their time.

### 2.4. Data Analysis

Data analysis was conducted using NVivo 14 software. Two researchers thematically analyzed the interviews to inductively generate codes [39]. A consensus coding process, where both researchers coded the same interview, was carried out for four transcripts to ensure consistency. The remaining transcripts were independently analyzed using a preliminary codebook generated during the consensus process. Weekly discussions were held between researchers to practice reflexivity and incorporate new themes or insights into the codebook. The process was iterative, and a detailed audit trail of coding decisions was kept throughout the analysis. Triangulation with field notes was used to ensure key themes were captured from each transcript.

Overarching themes within the codebook were considered in relation to the target behaviour (i.e., patient engagement in cancer prehabilitation) and categorized as barriers or facilitators. These behavioural determinants were mapped to the TDF and COM-B model [40] and linked to intervention functions using the BCW [37]. Example quotations were selected for each determinant, as well as for patient recommendations for intervention content or delivery. Example quotations were copied verbatim from transcripts, except for the removal of repeated and filler words and author insertions to provide missing contextual information (indicated using square brackets).

### 2.5. Researcher Contributions and Reflexivity

The interview guide was developed by YYL, NS, AJH, and LY. Participant recruitment and interviews were conducted by two female researchers (YYL and HH). Codebook development and data analysis was done by HH, NS, and YYL. All researchers have prior experience in academic research environments, with LY and NS having prior experience working with patients on interventional studies related to cancer prehabilitation. The research team collaboratively communicated throughout data collection and analysis to ensure credibility and trustworthiness of the research findings.

## 3. Results

### 3.1. Participant Characteristics

A total of 34 men diagnosed with prostate cancer took part in qualitative interviews. Approximately one third of patients (10/34; 29%) were recruited from a Tai Chi prehabilitation trial (6 from intervention and 4 from control arm), and the remaining 71% had not been involved in any formal prehabilitation programming. The study sample included 15 patients who had undergone primary surgical treatment, seven who had undergone primary non-surgical treatment modalities, three who were scheduled for and awaiting non-surgical treatment, and nine who were in an active surveillance program. Among treated patients, 23% (5/22) had received more than one treatment modality (e.g., combination androgen deprivation therapy + radiation, radiation following surgery, etc.). Although all participants were initially diagnosed with localized prostate cancer, one patient had metastatic disease at the time of their interview. Details on participants’ sociodemographic and clinical information are reported in Table 1.

### 3.2. Barriers and Facilitators to Patient Engagement in Prehabilitation

**Figure 1:**
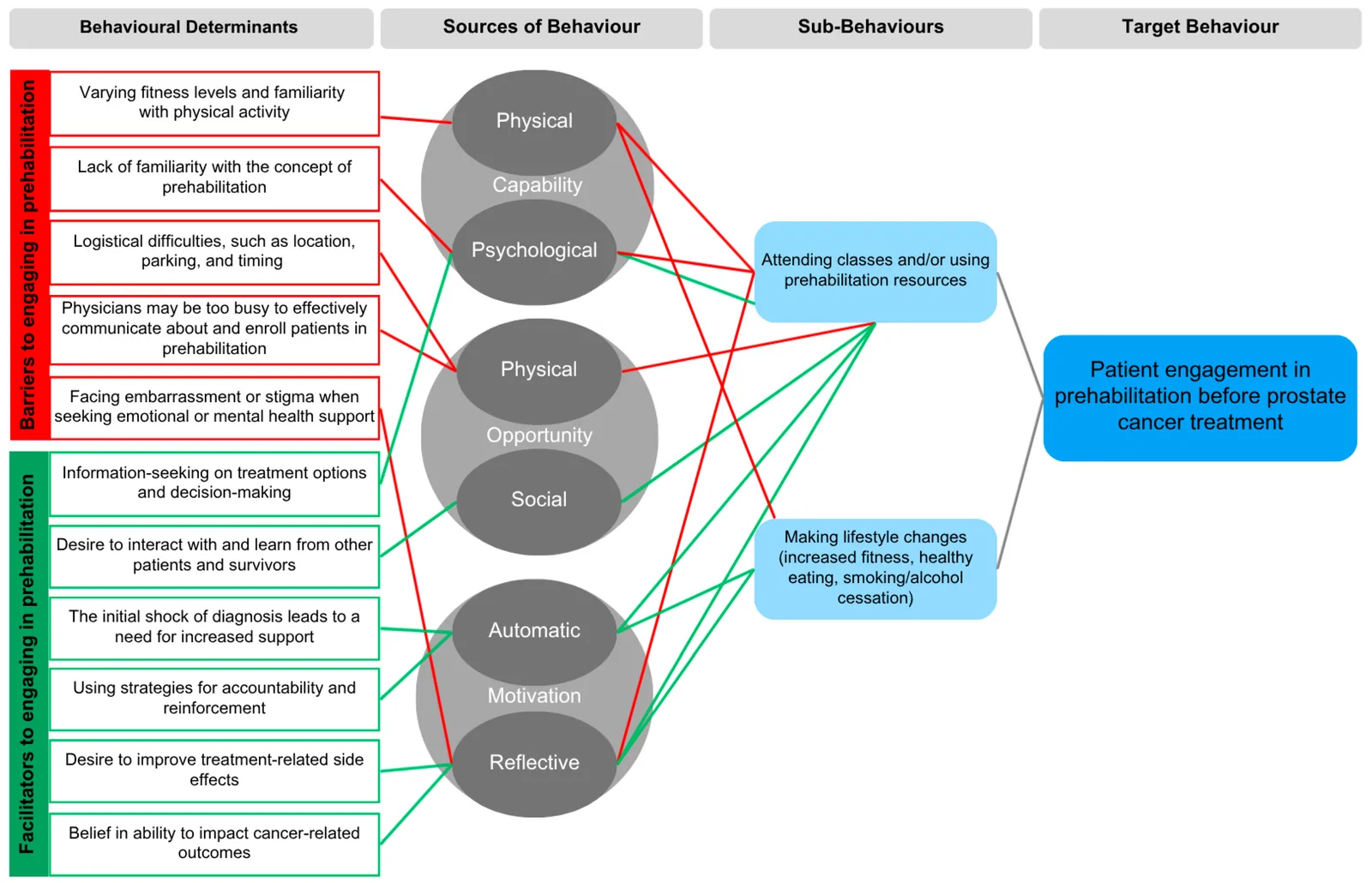
Barriers and facilitators to patient engagement in cancer prehabilitation mapped to sources of behaviour. Behavioural determinants were identified from thematic analysis and mapped to COM-B sources of behaviour using domains of the TDF [40]. Relationships between determinants and TDF domains are presented in Table 2, along with recommended techniques for behaviour change. Abbreviations: COM-B: Capability, Opportunity, Motivation-Behaviour; TDF: Theoretical Domains Framework.

**Figure 2:**
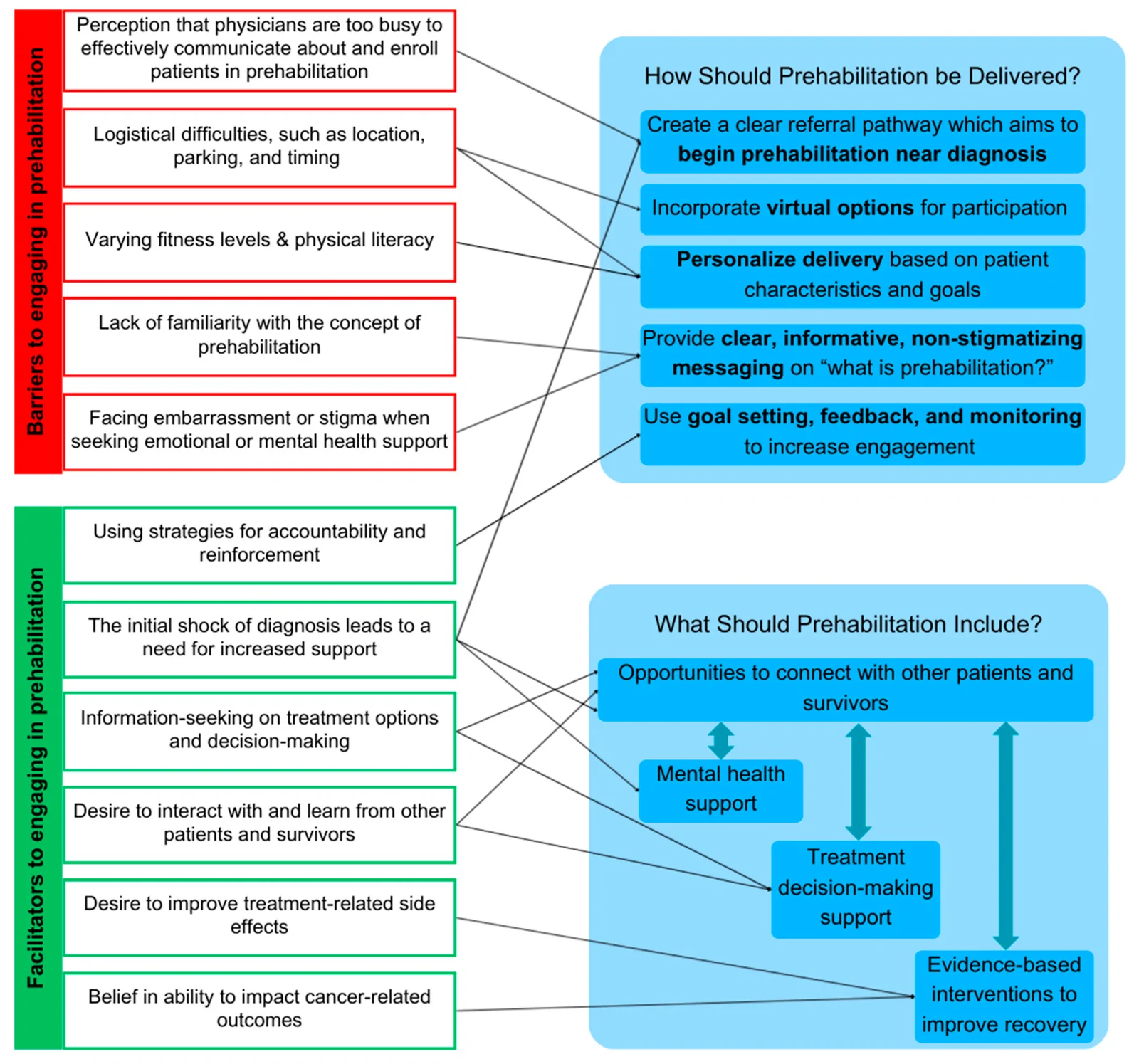
Barriers and facilitators to patient engagement in cancer prehabilitation linked to recommendations for intervention design. Behavioural determinants were identified from thematic analysis with recommendations for prehabilitation design informed by intervention functions from the behaviour change wheel (BCW) [37], in combination with direct recommendations on program content and delivery from participant interviews (Table 3).

We identified five barriers and six facilitators to patient engagement in cancer prehabilitation. The barriers and facilitators with links to the theoretical frameworks are presented in Table 2, along with example quotations. Barriers predominantly related to capability and opportunity sources of behaviour, while facilitators predominantly related to motivational sources of behaviour (Figure 1). Barriers were most relevant to informing delivery strategies for prehabilitation interventions, with themes related to messaging, personalization, and logistics. Meanwhile, facilitators were predominantly relevant to informing intervention content, with themes related to incorporating mental health and peer support, treatment education, and evidence-based interventions to improve treatment-related side effects (Figure 2). Specific patient recommendations on prehabilitation design are presented in Table 3.

#### 3.2.1. Capability

In general, participants reported relatively high levels of physical activity (Table 1), with most participants describing themselves as active and healthy. Despite generally expressing satisfaction with their fitness and lifestyle choices, they highlighted changes in their health as they aged and faced injuries or comorbidities. Additionally, participants highlighted that many patients diagnosed with prostate cancer have varying levels of familiarity with physical activity, which may be a barrier to participating in prehabilitation exercise (Table 2(a)).

*“It would be good to know physically what you need to do to keep yourself going and prepare yourself… [but] there are a lot of people involved with different conditions that some of them can do something about, some can’t. The whole variety of people go through. I don’t know if everyone can do what needs to be done.”*

Participant 16, age 71

Participants suggested that addressing these differences in physical capability could be accomplished by designing a program with older men in mind and personalizing the program to offer different options based on patients’ fitness and comfort with exercise.

*“I certainly wouldn’t be against going to somebody that says, ‘hey, let us come in for a day…we’ll see what equipment you’ve got and what we can do’ and say, ‘here’s what would be a good thing.’ I could still do that.”*

Participant 19, age 76

Our study identified psychological capability, particularly the domain of knowledge, as both a barrier and facilitator to patient engagement in cancer prehabilitation. More specifically, patients’ lack of familiarity with prehabilitation was a barrier (Table 2(b)), while their desire to gain more knowledge about their diagnosis and treatment was an important facilitator (Table 2(c)).

*“You’re trying to search for information and ideas and so I think you’re inclined to do [prehabilitation].”*

Participant 15, age 76

Although most participants did not have familiarity with the term prehabilitation, when introduced and explained, participants generally expressed interest in accessing prehabilitation resources or programs, highlighting the need for strategies aimed at increasing awareness and knowledge. Furthermore, many participants expressed that they faced difficulties making choices regarding their treatment plan, largely based on knowledge gaps surrounding their diagnosis and treatment. These patients generally expressed a desire to be more informed during the treatment decision-making and preparation phases, making the provision of information a facilitator to engaging in cancer prehabilitation.

#### 3.2.2. Opportunity

Participant interviews revealed two key barriers related to physical opportunity: perceived physician busyness and program logistics (Table 2(d,e)). Despite expressing that urologists were trusted sources of information whose opinions and recommendations were valued, participants cited concerns regarding their time constraints and competing care priorities (Table 2(d)). This was seen as a potential barrier to effectively enrolling patients in and communicating with them about cancer prehabilitation.

*“We have limited contact and these professionals, like the surgeon, they are I think very busy people. I don’t think that they have the time to hand hold or discuss…they don’t have a lot of time to spend on that type of thing.”*

Participant 20, age 72

To address this concern, participants felt that detailed information about prehabilitation should be provided by another source, for instance from patients who have been through the process or program facilitators who can provide detailed insights on what is offered and the benefits of participating.

Regarding logistical considerations, location was the most frequently identified barrier to attending, followed by timing (i.e., time of day or day of week). Participants cited distance to possible prehabilitation venues as a barrier and commonly referred to expensive or inconvenient parking as a significant deterrent to attending. Regarding scheduling of the program, participants identified work and conflicting appointments as interfering with their ability to attend. Commonly, patients felt that the provision of virtual programming was an acceptable method to reduce these barriers; however, a majority endorsed a hybrid program, with options for in-person participation due to (1) creating better social connections in-person, (2) lower levels of engagement during virtual sessions, and (3) lack of comfortability with technology among older patients.

*“Virtual, I think, allows you to have more freedom, more- well- easier access. It’s a little more limiting in terms of when you can dialogue compared to just being face-to-face.”*

Participant 26, age 67

Social opportunity was an important facilitator to engage patients in cancer prehabilitation, as a vast majority of participants expressed a desire to connect with and learn from other patients and survivors (Table 2(f)). Opportunities for social connection were seen as valuable throughout various components of prehabilitation, not only through peer support groups, but also as a part of treatment decision-making, mental preparation for treatment, and attending exercise sessions or classes (Table 3). Regarding the organization of connecting patients with peers, participants highlighted the importance of “building networks” which could involve either one-on-one connections or small groups, depending on individual preferences.

*“I was motivated because I knew I would be connected to other people.”*

Participant 5, on joining a prehabilitation trial, age 68

#### 3.2.3. Motivation

Participants shared the experience of facing shock and emotional distress in response to receiving a cancer diagnosis (Table 2(g)). The stress surrounding diagnosis was relevant to engaging in prehabilitation programming as participants expressed interest in accessing resources to support their mental wellbeing during this time. More specifically, many participants reflected on high levels of uncertainty in the time surrounding diagnosis, as they were often unsure about what the next steps and timelines were, which options were best for them, and what their outcomes would be.

*“Especially in the pre-operation stage, it becomes a bit of a black box… You have a bit of an idea, but you haven’t really absorbed it as far as the surgery itself, and what’s going to happen after the surgery, and what does that mean, and where are you going to go?”*

Participant 3, age 68

In another domain of automatic motivation, participants referenced strategies of reinforcement and accountability as a potential facilitator to engaging in cancer prehabilitation (Table 2(h)). Several participants referred to other areas of their life in which they used notification-based positive reinforcement to motivate lifestyle changes; some examples provided by participants included fitness tracking devices and educational mobile applications that monitored their progress, prompted them with notifications or challenges, and made them feel accomplished while completing tasks. This was analogized to strategies that would be useful for maintaining patient interest and sustaining behaviour change during cancer prehabilitation. Participants suggested that this could be implemented in prehabilitation interventions through repeated follow-up and progress-tracking by program facilitators, or the use of tools such as checklists, mobile applications, or updates/reminders delivered by regular emails, text messages, or phone calls.

Participants identified reluctance talking about mental health concerns as a potential barrier to engaging in certain prehabilitation components (Table 2(i)); this was commonly referenced in relation to participants’ perceptions of masculinity. Participants made recommendations for incorporating these concerns into program design by: (a) avoiding the use of potentially stigmatized terms in program messaging; for example, an emphasis on “needing help” may invoke feelings of avoidance or irrelevance among patients, and (b) using other components, such as mind-body activities, to address stress and anxiety without patients having to engage in a “mental health” program.

*“As a guy, my first thought is: ‘no, I don’t need help.’ That’s my go-to. And I [would be more likely] to go if it’s [presented as] more of a suggestion than an invitation.”*

Participant 27, age 58

In the pre-treatment phase, participants also commonly faced worries about post-treatment incontinence and erectile dysfunction and were in search of ways to reduce their risk of facing these issues (Table 2(j)). Participants highlighted that the desire to improve treatment-related side effects could be leveraged to engage patients in an evidence-based program designed to improve recovery. Many participants highlighted that presenting detailed and convincing evidence on the benefits of cancer prehabilitation would be a strong motivator for them to become involved.

*“I would want to look at the information that’s been presented and be assured that it has a positive outcome and that it’s not, for example, something that would have had a positive outcome if I started doing it when I was 20 years old–that it would have a positive outcome starting it at this stage. And then, yes, I would definitely want to [join a prehabilitation program].”*

Participant 23, age 73

This was additionally related to participant beliefs that their lifestyle choices could have an impact on cancer-related outcomes (Table 2(k)). Patients who had made changes to “get in better shape” before treatment commonly expressed that this led to positive impacts on their ability to handle the treatment and recovery phases. Many participants also highlighted that they believed both physical and mental preparation would be useful to help patients come through their treatment with better outcomes.

*“The better physical shape you are, the better off you are. But I think the mental side of it was really huge because– like all things–[if] you have that positive mental attitude, you can meet a lot of these challenges.”*

Participant 2, age 64

### 3.3. Interest in Participating in Cancer Prehabilitation

Among participants who had not been involved in any prior prehabilitation research (24/34), 16 (66.7%) expressed that they would have been interested in participating in prehabilitation, three (12.5%) expressed that they may have participated depending on the type of offerings, and five (20.8%) expressed that they would not have been interested in prehabilitation. Among participants who expressed lack of interest in prehabilitation, their disinterest was generally described as being related to personal characteristics, such as “not being a joiner” or preferring to do things on their own.

## 4. Discussion

Through in-depth qualitative interviews with men with prostate cancer, our investigation identified determinants of patient engagement in cancer prehabilitation, providing information on current practice gaps and recommendations for incorporating patient perspectives into the design of prehabilitation interventions. Across treatment types, participants generally expressed a desire to learn about and participate in behaviours that could improve their physical and mental wellbeing during the pre-treatment phase of their cancer journey. Barriers to engagement, including lack of familiarity with prehabilitation, limited availability, and reluctance to seek help, are important considerations when designing program delivery strategies. Identified facilitators to patient engagement pointed to a comprehensive design containing multiple components such as treatment education, peer support, and evidence-based strategies for improved recovery that can be personalized to patients’ specific needs. Consideration of these barriers and facilitators when designing prehabilitation interventions may help improve uptake of and meaningful engagement from patients during the often overwhelming time between cancer diagnosis and treatment.

Several prior studies have investigated the barriers and facilitators to patient engagement in surgical cancer prehabilitation. In line with our findings, a thematic synthesis of 26 studies highlighted the importance of providing personalized care which incorporates social support, addresses the emotional impact of diagnosis, and leverages patient desire to improve long-term health [43]. The consistency of behavioural determinants identified in our study with those reported in surgical populations suggests that many core elements of surgical prehabilitation programs may be adaptable to patients receiving other treatment types.

While there is a relatively large body of literature on surgical prehabilitation, to our knowledge, our study is the first to investigate patient perspectives on cancer prehabilitation across treatment types. Although prior studies have investigated patient perspectives on specific pre-treatment interventions, such as treatment education [44,45,46], our investigation is unique in exploring multiple intervention components to create priorities for designing a comprehensive prehabilitation program. Evidence from surgical cancer prehabilitation has shown that effective implementation depends on intervention design grounded in collaboration and a focus centering on the patient voice [47]. Despite growing recognition of its importance, the patient perspective has not been consistently or systematically incorporated into the development of healthcare interventions [48,49]. By integrating patient involvement as a key component of intervention development, our study advances the design of prehabilitation interventions which consider and incorporate the needs of the end-user group and identify meaningful priorities for improving patient care in the pre-treatment period.

Distinct from the delivery of surgical prehabilitation, our study identified the time between diagnosis and treatment decision-making as an important gap when engaging patients in prostate cancer prehabilitation. Patients expressed a need for increased support to address stress and uncertainty during this period and highlighted informational, psychological, and social needs which could potentially be addressed by a comprehensive prehabilitation intervention. Traditional models of prehabilitation, which specifically target surgical patients, are inherently delivered after patients have already decided on their treatment modality and much of this anxiety and uncertainty may be less prominent. Although other psychosocial resources may be available to newly diagnosed patients, our findings suggest that the incorporation of these types of interventions into a comprehensive prehabilitation program may help to encourage patient engagement and may be most effective when delivered before patients have made a treatment decision. As such, our findings highlight the potential for incentivizing recruitment and interest in prehabilitation activities by delivering cancer prehabilitation earlier in the cancer care continuum.

While most participants expressed interest in prehabilitation, several were unfamiliar with the concept or described themselves as being uninterested in participating. These findings highlight the need for clearly described programming with personalized engagement options to accommodate differing preferences for social interaction. Although social connection was an important motivator for some patients, others preferred more individual, independently directed activities. Virtual and home-based interventions have demonstrated feasibility and acceptability in prehabilitation settings [30,50] and may help address logistical barriers while better meeting the needs of patients who prefer independent participation. Hybrid programming, offering both in-person and virtual options, may therefore provide a flexible approach that accommodates the range of patient preferences for participation, and was commonly endorsed as an ideal delivery model by participants in our study.

Although participants generally expressed a willingness to participate in activities aimed at improving post-treatment outcomes, few interventions have demonstrated effectiveness in mitigating the side effects of prostate cancer treatment. In surgical settings, exercise prehabilitation for patients with urologic cancer has been shown to result in improved physical fitness and quality of life, but there is little to no evidence for a reduction in postsurgical complications [19]. Beyond surgical settings, the evidence is even more limited [10]. This evidence gap is particularly important as our investigation found that participants’ motivation to engage in prehabilitation interventions was largely influenced by whether there were proven benefits for improving treatment-related side effects, specifically regarding sexual function and urinary incontinence. Future studies assessing the efficacy and effectiveness of cancer prehabilitation interventions should measure and target the outcomes most meaningful for patients, with a focus on reducing the severity of treatment-related side effects and managing these side effects. Additionally, greater research is needed on prehabilitation interventions for non-surgical patient populations. The provision of this evidence and information would be useful to improve treatment preparation and patient motivation to engage in prehabilitation.

### Strengths and Limitations

Our findings were developed using qualitative methods centered on maintaining trustworthiness and credibility, supporting the study’s internal validity. The concordance of identified themes with previous literature suggests that our recommendations may be applicable to broader contexts, beyond patients receiving care in Alberta, Canada. However, the transferability of these findings to other settings or cancer types should be carefully considered. While certain themes, such as the emotional impact of diagnosis and desire for social support, are likely relevant across cancer types [51,52]. some themes may require further investigation. For example, the stigma surrounding mental health support discussed in this investigation was largely related to participant-identified perceptions of masculinity and may present differently across cultures or among women or mixed gender groups. Furthermore, the outcomes of importance identified in our study are primarily related to urinary or bowel incontinence and sexual dysfunction; the ideal targets of prehabilitation for other cancers require consideration of outcomes specific to the cancer type and its treatments. Finally, participants in our study had relatively high socioeconomic status, with the majority reporting a bachelor’s or graduate degree and household income ≥ CAD $100,000. This may limit the transferability of our findings to the broader population of men with prostate cancer, particularly those with lower socioeconomic status or who experience greater social and structural barriers to accessing care.

While a strength of our study is the representation of patient perspectives, the current study did not include multi-end-user groups, such as patient caregivers, healthcare providers, and program administrators. Future investigations to incorporate these perspectives will be conducted to strengthen the feasibility and impact of intervention design and implementation. Finally, our investigation aimed to explore and summarize key themes relevant across prostate cancer treatment modalities. While these findings may be useful for promoting patient engagement, there is significant heterogeneity of patient experiences and priorities across treatment types. The design of targeted prehabilitation interventions should incorporate treatment-specific considerations, in addition to considering the broader determinants of engagement identified in our study.

## 5. Conclusions

By systematically incorporating patient perspectives and leveraging behaviour change principles, this study provides recommendations to improve uptake and impact when designing prehabilitation interventions for prostate cancer patients. Prehabilitation before cancer treatment was seen as useful and important to patients with prostate cancer across treatment pathways. Future implementation of prehabilitation in prostate cancer care should incorporate delivery strategies that provide timely, personalized, and evidence-based prehabilitation interventions to address current practice gaps and increase patient engagement. Despite interest in participating in prehabilitation, there is limited evidence for interventions that can mitigate the range of short- to long-term side effects associated with prostate cancer treatment. To motivate patients to engage in prehabilitation, further research is needed to identify evidence-based prehabilitation interventions targeting outcomes that are important for prostate cancer patients, such as post-treatment sexual and urinary function.

## Figures and Tables

**Table 1:** Demographic and clinical characteristics of patients with prostate cancer who participated in qualitative interviews (*n* = 34).

Variable	Number of Participants (%)
**Mean Age**	66.74 ± 5.34 years (range 55 to 76)
**Racial or Ethnic Identity**	
White	28 (82.4%)
Asian	4 (11.8%)
Black	2 (5.9%)
**Treatment Modality ^1^**	
Surgery	15 (44.1%)
Radiation Therapy	9 (26.5%)
Hormone Therapy	4 (11.8%)
Brachytherapy	2 (5.9%)
Cryotherapy	1 (2.9%)
Active Surveillance	9 (26.5%)
**Tumor Grade ^2^**	
1	7 (20.6%)
2	21 (61.8%)
≥3	4 (11.8%)
**Highest Level of Education**	
High school	3 (8.8%)
Post-secondary certificate or diploma	6 (17.6%)
Bachelor’s degree	14 (41.2%)
Graduate degree	11 (32.4%)
**Household Income ($CAD)**	
Under 50,000	1 (2.9%)
50,000–74,999	4 (11.8%)
75,000–99,999	6 (17.6%)
≥100,000	18 (52.9%)
Not Reported	5 (14.7%)
**Working Status**	
Retired	20 (58.8%)
Working	13 (38.2%)
Unemployed	1 (2.9%)
**Self-Reported Physical Activity Level ^3^**	
<7 h per week	8 (23.5%)
7–14 h per week	17 (50.0%)
>14 h per week	9 (26.5%)
**Involvement in Prior Prehabilitation Research**	
Not involved in any formal programming	24 (70.6%)
Recruited from prehabilitation pilot trial	10 (29.4%)
Tai Chi intervention arm	6 (17.6%)
Usual care control arm	4 (11.8%)

^1^ Percentages exceed 100% as patients could have undergone multiple treatments; participants who were scheduled for and awaiting a specific treatment modality were included in counts; ^2^ Missing for *n* = 2 participants who did not consent to chart review of clinical information; ^3^ Estimated in hours per week, including light physical activity such as walking or stretching.

**Table 2:** Behavioural determinants of patient engagement with combined links to the COM-B model, TDF domains, and general recommendations for cancer prehabilitation design.

Barrier or Facilitator	COM-B Component *[TDF Domain]*	Example Quotations	Intervention Functions	Behaviour Change Techniques	Recommendations for Prehabilitation Design
**(a) Varying fitness levels and physical literacy** (barrier)	Physical Capability *[Physical skills]*	*“If somebody hasn’t done any physical exercise their whole life and now they’re 70 and they’re diagnosed with prostate cancer, you have to be realistic.”* Participant 15, age 76 *“Once you’re over 65, I noticed personally physiological changes in my body and my ability to maintain fitness and things like that.”* Participant 22, age 68	− Enablement − Training	− Instruction on how to perform the behaviour − Graded tasks	Offer individualized coaching and assessment Create accessible physical activity programs that are designed to accommodate individuals with various fitness levels
**(b) Lack of familiarity with the concept of prehabilitation** (barrier)	Psychological Capability *[Knowledge]*	*“It’s a new word. I think you just invented it- called prehabilitation. If I look it up in Webster’s dictionary, I don’t think I’ll find that word.”* Participant 19, age 76	− Education	− Information about health consequences − Credible source	Define and describe prehabilitation in all communication about programs
**(c) Information-seeking on treatment options and decision-making** (facilitator)		*“In the earliest stages, just being told you have cancer doesn’t sit well… I mean that early diagnosis just says, ‘you have prostate cancer.’ Now, it’s like, okay, I know nothing about cancer.”* Participant 1, age 60 *“The information session had one slide at the end of a 50 slide presentation that said you should think about losing weight and stopping smoking and not drinking and other more general things. Well, I guess I’d like to have a much more specific conversation on that.”* Participant 24, age 70 *The more information you have, the more times you can listen to what’s going on, it takes the black box out of it.”* Participant 3, age 68	− Education	− Information about health consequences − Instruction on how to perform a behaviour − Reduce negative emotions	Design and provide educational resources to support patients in treatment decision-making Prehabilitation programs should begin before patients decide on their treatment
**(d) Perception that physicians are too busy to effectively communicate about and enroll patients in prehabilitation** (barrier)	Physical Opportunity *[Environmental context and resources]*	*“The medical specialists, they’re not very good at explaining things because they’re looking at their watch and they’re talking to you, and they know that they got somebody [else coming].”* Participant 20, age 72 *“It would be nice to talk to the doctor about [prehabilitation], but they’re pretty busy…That’s just adding one more thing to their plate.”* Participant 32, age 65	− Enablement − Modelling	− Demonstration of the behaviour − Prompts/cues − Action planning	Create a clear and easy pathway for referral Model behaviours of urologists who are willing and able to teach their patients about prehabilitation
**(e) Logistical difficulties, such as location, parking, and timing** (barrier)		*“I think there was even some exercise classes that were available, but they were right in the middle of work, and I worked late…if you’re older and retired, then you can take advantage of those sorts of things a bit more.”* Participant 30, age 65 *“You’d want it to be a nice venue where people want to go, and preferably with free parking. That’s always an irritant when you have pay for parking.”* Participant 25, age 66	− Environmental restructuring − Enablement	− Restructuring the physical environment	Offer virtual program components to increase accessibility and convenience Consider patients who may be working or have other commitments when scheduling programs
**(f) Desire to interact with and learn from other patients and survivors** (facilitator)	Social Opportunity *[Social influences]*	*“For me to feel motivated, or to feel the willingness to move forward in that avenue would be if there were opportunities to dialogue”* Participant 26, age 67 *“I feel like my stress went from zero to 100 in a fairly short period of time. But yeah, there wasn’t really anything [available], so I had to manage my stress. I had to manage my stress…Before the surgery it would have been nice to have something”* Participant 31, age 58	− Environmental restructuring	− Restructuring the social environment − Social support	Create programs to facilitate patient connection with peers while providing options for both group and one-on-one interactions
**(g) The initial shock diagnosis leads to a need for increased support** (facilitator)	Automatic Motivation *[Emotion]*	*“You don’t have cancer one day and the next day somebody tells you have cancer. And that was a huge turning point for me where you just felt lost, like, what are you going to do?”* Participant 1, age 60 *“When you got cancer, you do go into a vacuum in your own mind… and it works, that the patient sits in this vacuum going to all the bad places in his mind”* Participant 14, age 68	− Enablement − Environmental restructuring	− Reduce negative emotions − Conserving mental resources	Approach patients about joining prehabilitation programs as early as possible in the care continuum (near diagnosis)
**(h) Using strategies for accountability and reinforcement** (facilitator)	Automatic Motivation *[Reinforcement]*	*“It’s almost like a game. They give you points and you kind of challenge yourself… it makes it fun and also I want to challenge myself to do better at it. So, if there was something like that with the diet and the fitness and the drinking and the whatever else it was, little goals or the things like that, challenges to do.”* Participant 21, age 67	− Persuasion − Incentivization	− Goal setting − Feedback on behaviour − Prompts/cues − Incentive (behaviour)	Use notifications and challenges to help patients stay motivated Have program facilitators follow up with patients about their progress and goals
**(i) Facing embarrassment or stigma when seeking emotional or mental health support** (barrier)	Reflective Motivation *[Social role and identity]*	*“I think guys are hesitant to think that they need mental health support…I always use this term Superman-type mentality, and I think that would be a barrier to joining something”* Participant 2, age 64 *“People aren’t going to talk about their- especially guys- they aren’t going to talk about their concerns and worries and fears as much. But it does help a bit.”* Participant 16, age 71	− Education − Persuasion	− Remove aversive stimulus − Reduce negative emotions − Social comparison	Present programs in a non-stigmatizing, normalizing way which encourages participation Offer program components such as social support and mind-body exercises which can help address mental health concerns
**(j) Desire to improve treatment-related side effects** (facilitator)	Reflective Motivation *[Goals]*	*“If they said your chances of not having incontinence for a period of time [are lower], that would have been a big motivating factor that I better get serious about doing some exercises.”* Participant 10, age 68 *“I wasn’t worried about the procedures. It’s the after consequences of that—what the effects of the treatment might be was my biggest fear.”* Participant 18, age 55	− Education	− Information about health consequences − Feedback on outcomes of behaviour	Design a program which is related to patient goals and priorities, with outcomes specific to cancer type and treatment type Create messaging or reminders about the potential benefits of prehabilitation to help motivate patients to enroll and engage
**(k) Beliefs that lifestyle changes can impact cancer-related outcomes** (facilitator)	Reflective Motivation *[Belief in consequences]*	*“I think that from a health goal perspective, definitely a reduction in weight and improvement in fitness would be desirable just in recovering from any type of therapy I might have, whether it’s surgical or radiation or whatever it is.”* Participant 22, age 68, active surveillance *“What you have to go through is serious and if you have better mobility going in, you’re probably going to have better recovery coming out.”* Participant 34, age 67	− Persuasion	− Goal setting − Commitment − Discrepancy between current behaviour and goal − Information about health consequences	Use goal setting, education, and commitment to reinforce the importance of prehabilitation activities Create messaging or reminders about the potential benefits of prehabilitation to help motivate patients to enroll and engage

**Table 3:** Specific patient recommendations to address barriers and facilitators to engagement in cancer prehabilitation.

Patient-Recommended Behaviour Change Techniques	Example Quotations	Barrier(s) or Facilitator(s) Being Addressed
Have realistic physical activity goals and peer leaders or role models who can demonstrate physical activity participation and benefits	*“I wouldn’t want some gung-ho, 21-year-old kinesiology graduate to tell me ‘Oh, you got to do 50 push-ups’… you got to look at your target audience which in this case would be men over 50 and potentially in their 60s, right? So, a lot of us can’t do our 50 push-ups.”* Participant 27, age 58 *“A lot of these personal trainers are in the greatest shape ever and that’s not always the motivator for someone that’s 250 pounds. The motivator is this guy that’s 66 years old and was 50 pounds overweight, and now he isn’t, and he’s come through it in less than a year and it’s like nothing happened.”* Participant 8, age 66	Varying fitness levels and physical literacy (barrier)
Tailor physical activity components so they can be modified to different variations	*“The challenge is when people have limitations…But I’ve seen a few of the [exercise] classes where the instructor has been very good at modifying things during the class, and so it’s good to see that.”* Participant 12, age 70	
Connect patients with survivors who can provide them with a personal perspective on treatment options	*“I phoned a couple of guys that have had this cryo-surgery before…They were more than helpful to answer any of the questions or tell me about their procedures and stuff like that. So that was a big help to [make a decision], just being able to talk to somebody who had gone through the procedure.”* Participant 17, age 67 *“You get a much a much more personal perspective from someone that can say, ‘Yeah, but…’ or ‘Have you thought of this?’ or ‘Yes, I went through this, and this is what it looks like after.’ I think that would be really a beneficial thing.”* Participant 26, age 67	Information-seeking on treatment options and decision-making (facilitator) Desire to interact with and learn from other patients and survivors (facilitator)
Reach out to newly diagnosed patients to offer mental health resources and support	*“If immediately after you had the consult with the doctor, you could even spend 15 to 20 min with somebody on the mental health side saying ‘you’ve just heard some really bad news. How are you processing it?’—that type of a discussion. I think that would have been welcomed. It doesn’t exist, but it would have been welcome.”* Participant 28, age 59	The initial shock diagnosis leads to a need for increased support (facilitator)
Use messaging which avoids making patients feel like they are abnormal or weak	*“I think if you labeled it more as a support group to figure out how to help your people close to you handle the situation, more so than yourself… then I would be more motivated to jump in versus something that says, ‘you need some mental health help.’ Well, that just sounds like ‘you’re nuts’ and that would kind of push me away from it.”* Participant 2, age 64	Facing embarrassment or stigma when seeking emotional or mental health support (barrier)
Organize program components that encourage informal peer support which can be an alternative way for patients to address their anxieties or concerns	*“We’re quite skeptical of mental treatment. We’re also uncomfortable with other people in the same group openly discussing it for mental purposes. But we’re not afraid to mention, ‘Hey, how are you doing?’, ‘How’s it going?’, ‘Did you make your treatment decision?’, ‘Why did you do that?’ as a general conversation once you’re comfortable with knowing a person… [during] Tai Chi that was happening, but in a way incognito to the other activity.”* Participant 5, age 68 *“If you’re going to do some physical stuff it’s always good to get a bunch of old guys together so they can chat and joke about it, so yeah, there would be a social aspect to that. I think that would be very good…It never hurts to get a bunch of guys together to talk about what’s going on.”* Participant 30, age 65	Desire to interact with and learn from other patients and survivors (facilitator) Facing embarrassment or stigma when seeking emotional or mental health support (barrier)
Present patients with specific evidence about how prehabilitation could benefit them	*“I think prehabilitation has to be presented as a ‘this is in preparation’… For me, I am a stat[istic]s guy to a certain degree, so if I had ‘this has helped this many people this way’…if you can get the percentages sorted out, I think that would work really well.”* Participant 27, age 58	Desire to improve treatment-related side effects (facilitator) Beliefs that lifestyle changes can impact cancer-related outcomes (facilitator)

## Data Availability

The data collected and analyzed during this study are not publicly available due to participant privacy and confidentiality. De-identified participant transcripts may be made available from the corresponding author on reasonable request.

## Supplementary Materials

The following supporting information can be downloaded at: https://www.mdpi.com/article/10.3390/curroncol33090564/s1, File S1: Standards for Reporting Qualitative Research (SRQR) checklist. File S2: Informed Consent Form. File S3: Semi-Structured Interview Guide.

## Abbreviations

BCW—Behaviour Change Wheel; COM-B—Capability, Opportunity, Motivation—Behaviour; TDF—Theoretical Domains Framework.
