# Supplementary material for: Patient Perspectives on Prostate Cancer Prehabilitation: A Qualitative Study Using the Behaviour Change Wheel to Investigate Barriers and Facilitators to Engagement
Source: Curr Oncol. 2026 Sep 17;33(9):564. doi: 10.3390/curroncol33090564 (PMC13605913; doi:10.3390/curroncol33090564)
Supplement: Supplementary file 1 [file curroncol-33-00564-s001.zip › File S2_Informed Consent Form.pdf]

Informed Consent Form

STUDY TITLE: The PEAK program: Development, Implementation, and Evaluation of a Cancer Prehabilitation Program to Enhance Knowledge, Improve MentAl Health and Keep Up Physical Function

SPONSOR: Canadian Cancer Society (CCS)

ETHICS: Ethics ID #: HREBA.CC-22-0362

INVESTIGATORS:

Principal Investigator:

Dr. Lin Yang, PhD
Department of Cancer Epidemiology and Prevention Research
Cancer Care Alberta
Email: lin.yang@cancercarealberta.ca

Co-investigator: M. Eric Hyndman, MD. Prostate Cancer Centre/University of Calgary.

External Collaborators (Co-Investigators): Ashely Housten and Anna Baumann, Washington University School of Medicine, US.

Collaborators: Holly Bradley, Sara Schneideman, CCS; Dominic Sanford, Melanie Koenen, Chao Cao, Siobhan Sutcliffe, Adetunji Toriola, Washington University School of Medicine, US.

BACKGROUND

Prostate cancer is the most commonly diagnosed non-skin cancer in Canadian men, with 1 in 9 expected to be diagnosed with prostate cancer in their lifetime. Based on the stage of the cancer, age and general health, there are several therapy options available for treating prostate cancer. The first-line prostate cancer treatment options can range from active surveillance, surgery, radiation therapy, hormone therapy, and chemotherapy. Active surveillance can avoid immediate treatment related side-effects by postponing active treatment to a later time. Active treatment options, such as surgery, radiation therapy, hormone therapy, is each associated with unique treatment side-effects and psychosocial distresses that are related to cancer diagnosis, treatment decision, recovery, and treatment side-effects. For this reason, experiences undergoing cancer treatment and quality of life after treatment have become important factors in determining patients' satisfaction with their care. Therefore, interventions that improve psychological and physical health before cancer treatment are needed to provide the necessary tools and support for prostate cancer patients becoming better prepared for cancer treatment.

Prehabilitation, mental and physical preparation, is a concept emerged in the surgical setting, which helps to better prepare people for the stress of surgery and help them recover faster after surgery. The value of prehabilitation is gradually being recognized in cancer care as a mean to prepare patients for

other harsh cancer treatment modalities such as radiation therapy, hormone therapy and chemotherapy. However, there is no conceptual model available to guide the development, implementation, and evaluation of prehabilitation programs for cancer patients. We propose an evidence-based, theory driven approach to co-develop a logic model with patients and healthcare providers to guide the design, implementation, and evaluation of cancer prehabilitation programs.

The Health Research Ethics Board of Alberta – Cancer Committee (HREBA-CC), which oversees the ethical acceptability of research involving humans, has reviewed and granted ethics approval for this study.

WHY AM I BEING ASKED TO TAKE PART IN THIS RESEARCH STUDY?

You are being invited to participate in this research study because you have been diagnosed with prostate cancer and have gone through the treatment decision. You may have or have not undergone the treatment.

The purpose of the study is to understand prostate cancer patients' perspectives of what would make a safe, effective, and relevant prehabilitation program so as to design, implement, and evaluate a prehabilitation program for prostate cancer patients in the future. The naming of the prehabilitation program is the **PEAK** program, which stands for A cancer **P**rehabilitation program to **E**nhance knowledge, improve ment**A**l health and **K**eep up physical function.

This electronic consent form provides information about the study to assist you with making an informed decision. The researcher will discuss this study with you and will answer any questions you may have. You are encouraged to ask questions. When all your questions have been answered to your satisfaction, you can decide if you want to be in the study or not.

Taking part in this study is voluntary. You may choose whether or not you take part. If you choose to participate, you may leave the study at any time without giving reason or without penalty. Deciding not to take part or deciding to leave the study early will not result in any penalty or effect current or future care or employment.

If you decide to participate in this study, you will need to sign and date this consent form.

HOW MANY PEOPLE WILL TAKE PART IN THIS STUDY?

We plan to enroll approximately 50 people in this study. The primary caregiver of a patient participant is also eligible to participate. Caregiver participation is optional. Please contact the Principal Investigator or Study Coordinator (Lin Yang (XXX) XXX-XXXX or Study coordinator (XXX) XXX-XXXX) for more information on caregiver participation.

WHAT WILL HAPPEN DURING THIS STUDY?

This study will require your one-time participation in an interview, which will take approximately 60 minutes. In this interview, we will ask you about your experience between prostate cancer diagnosis and

treatment, your unmet needs and barriers to access information, support and resources to prepare for prostate cancer treatment. We will also ask you about your preferences for features and functionalities to include in a prehabilitation program that will help you to prepare for cancer treatment.

If you are interested, you will also be invited to participate in the planning group of the study. You will be given options to choose whether you would like to receive the invitation at the end of this consent form. Should you choose to participate, the involvement in the planning team is not to contribute research data as a study participant but engage the study as part of the research team to review, discuss and refine the materials, documents, protocol and manuals for the PEAK program.

At the completion of the study the researcher or study staff will be accessing your personal medical record to collect specific information related to your prostate cancer diagnosis (i.e. PSA results, Gleason score and pathology results). We are collecting this information to expand on and verify the information collected during the interview.

WHAT ARE MY RESPONSIBILITIES SHOULD I DECIDE TO PARTICIPATE IN THIS STUDY?

If you choose to participate in this study, you will be expected to attend a one-time a semi-structured virtual interview via zoom with a study team member. This interview is expected to take approximately 60 minutes. The interview will be video-recorded for transcription purposes and to observe our interaction together.

WHAT WILL HAPPEN IF I CHOOSE TO WITHDRAW FROM THE STUDY EARLY?

Participation is voluntary. You may withdraw your participation before, during and up until the end of the interview process. Should you wish to withdraw before the interview you may do so by contacting a research team member, either the investigator or the team member who coordinated the interview, and request to be removed from the research study.

If you want to withdraw from the study during the interview, please let the interviewer know and all documents and recordings associated with your interview will be destroyed.

The researchers may withdraw you from the study if 1) you are no longer considered eligible; or 2) your availability for the interview is not compatible with the research timelines.

WHAT ARE THE RISKS/DISCOMFORTS OF PARTICIPATING IN THIS STUDY?

This study poses minimal risk.

Participation in these interviews and research involves the probability and magnitude of possible harms implied by participation that is no greater than that encountered by participants in those aspects of their everyday life that relate to the research.

The data collected is not personal or sensitive and your confidentiality will be protected by the research group.

WHAT ARE THE POTENTIAL BENEFITS OF PARTICIPANTING IN THIS STUDY?

If you agree to participate in this study, there may or may not be a direct benefit to you. Participating in the development stage of a cancer prehabilitation program may help you to better understand your own barriers to accessing supportive care.

The results from the interviews will help us to develop the PEAK program to better serve the prostate cancer patients in the future.

HOW WILL MY PERSONAL INFORMATION BE KEPT PRIVATE?

If you decide to participate in this study, the study researchers and study staff will only collect the information they need for this study. The study team will do everything that they can to make sure that this data is kept private and confidential.

Records identifying you will be kept confidential to the extent permitted by the applicable laws, will not be disclosed or made publicly available, except as described in this consent document.
Authorized representatives of the following organizations may look at your identifiable medical/clinical study records at the site where these records are held for quality assurance purposes and/or to verify that the information collected for the study is correct and follows proper laws and guidelines:

- The Health Research Ethics Board of Alberta – Cancer Committee, which oversees the ethical conduct of this study;

Authorized representatives of the above organizations may **receive** information related to the study from your study records that will be kept confidential in a secure location and may be used in current or future relevant health research. Your name or other information that may identify you will not be provided (i.e., the information will be de-identified). The records received by these organizations will be coded with a number. The key that indicates what number you have been assigned will be kept secure by the researchers directly involved with your study and will not be released.

To ensure confidentiality and to provide a safeguard for participant identification, all participants will be assigned a study number. All the data collected in the interview will be filed and analyzed according to this number and any identifiable information will be removed from audio-recordings during transcription. Only the principal investigator and staff working with participants will have access to the participants' names and corresponding numbers assigned.

The study researchers will ensure that any personal health information collected for this study is kept in a secure and confidential location, a locked cabinet at the Holy Cross Centre, as also required by law.

The video/audio recordings will be stored in a secure location and viewed only by members of the research team. The recordings will be kept until they have been transcribed (turned into written records), and then they will be destroyed.

Computer systems at the study site are secure and encrypted. All data will be entered into a database on a password protected personal computer. Only the principal investigator and one study coordinator will have access to this password. The identity records and personal identifiers will be kept separately and securely from the collected data to avoid the possibility of directly linking personal identities to any collected data.

If the results of this study are published, your identity will remain confidential. It is expected that the information collected during the study will be used in analyses and will be published/presented to the scientific community at meetings and in journals.

Even though the likelihood that someone may identify you from the study data is very small, it can never be completely eliminated. Every effort will be made to keep your identifiable information confidential, and to follow the ethical and legal rules about collecting, using and disclosing this information.

Studies involving humans sometimes collect information on race and ethnicity as well as other characteristics of individuals because these characteristics may influence how people respond to different interventions. Providing information on your race or ethnic origin is required.

After the study is complete, we will still need to securely store your data that was collected as part of the study. Data and files collected will be stored for the legally required length of time of 10 years, at which point it will be destroyed.

WILL THERE BE COSTS INVOLVED WITH PARTICIPATING IN THIS STUDY?

No, you do not have to pay for anything as part of your participation in this study.

WILL I BE COMPENSATED FOR PARTICIPATING IN THIS STUDY?

As compensation for your time, you will be offered a $50 gift card for participating.

WHAT ARE MY RIGHTS AS A PARTICIPANT IN THIS STUDY?

You will be told, in a timely manner, about new information that may be relevant to your willingness to stay in this study.

You have the right to be informed of the results of this study once the entire study is complete. If you would like to be informed of these results, please contact the researcher.

Your rights to privacy are legally protected by federal and provincial laws that require safeguards to ensure that your privacy is respected.

By providing your consent you do not give up any of your legal rights against the hospital, researchers, sponsor, institutions or their agents involved for compensation, nor does this form relieve these parties from their legal and professional responsibilities.

IS THERE ANY CONFLICT OF INTEREST RELATED TO THIS STUDY?

There are not conflicts of interest relating to the conduct of this study.

WHO CAN I CONTACT FOR QUESTIONS RELATED TO THIS STUDY?

If you have questions about taking part in this study, you should talk to the Principal Investigator. This person is:

Lin Yang (Principal Investigator)	(XXX) XXX-XXXX
Name	Telephone

Or

Hannah Harsanyi (Research Assistant)	(XXX)-XXX-XXXX
Name	Telephone

Health Research Ethics Board of Alberta (HREBA):

If you have questions about your rights as a participant or about ethical issues related to this study and you would like to talk to someone who is not involved in the conduct of the study, please contact the Office of the Health Research Ethics Board of Alberta – Cancer Committee at:

Telephone: 780-423-5727 Toll Free: 1-877-423-5727

UNDERSTANDING AND SIGNATURES PAGE

	Yes	No
Do you understand that you have been asked to take part in a research study?	☐	☐
Do you understand why this study is being done?	☐	☐
Do you understand the potential benefits and risks/discomforts of taking part in this study?	☐	☐
Do you understand what you will be asked to do should you decide to take part in this study?	☐	☐
Do you understand that you are free to leave the study at any time, without having to give reason or without penalty?	☐	☐
Do you understand that we will be collecting information about you for use in this study only?	☐	☐
Do you understand that by signing this consent form that you do not give up any of your legal rights?	☐	☐
Do you feel that you had enough time and opportunity to consider the information provided to you by way of asking questions, having conversations with others and considering your options?	☐	☐

If a potential participant has answered "no" to any question above, please make sure to go over the relevant information with them until they do understand it. ***Only once they are comfortable with all the information can you accept their decision to participate in the study.***

AGREEMENT TO PARTICIPATE

Do you agree to participate in this study?

Clicking on the "agree" button below indicates that:

- You have read the above information
- You voluntarily agree to participate

☐ Agree

☐ Decline

Can we access your medical record to collect information about your prostate cancer diagnosis?

☐ Agree

☐ Decline

Do you consent to be contacted about participating in the PEAK program planning group as a patient advisor? You do not need to participate in the planning group and are free to withdraw at any time.

Clicking on the "agree" button below indicates that:

- You have read the above information
- You voluntarily agree to participate

☐ Agree

☐ Decline

CONSENT OBTAINED BY:

Name of Research Team Member	Date
