# Supplementary material for: Patient Perspectives on Prostate Cancer Prehabilitation: A Qualitative Study Using the Behaviour Change Wheel to Investigate Barriers and Facilitators to Engagement
Source: Curr Oncol. 2026 Sep 17;33(9):564. doi: 10.3390/curroncol33090564 (PMC13605913; doi:10.3390/curroncol33090564)
Supplement: Supplementary file 1 [file curroncol-33-00564-s001.zip › File S3_Semi-Structured Interview Guide.pdf]

Semi-Structured Interview Guide

Interview Section	***Question/Prompt***	***Theoretical Domain(s)***
Personal Reflection on Cancer Journey	**Can you walk me through your experience being diagnosed with prostate cancer?**<br>• How do you think your diagnosis impacts your daily life?<br>• How did you decide on your treatment plan?	 *Emotion; Social influences*<br><br>*Knowledge; Memory, attention and decision processes; Intentions; Goals*
	**How did you prepare for your treatment?**<br>• What did you find helpful in preparing for your treatment? E.g. any resources received, support from healthcare team, or peer support<br>• Did you receive any education about preparing for treatment, its side effects and potential outcomes?<br>• Looking back, is there anything you wished you knew ahead of time regarding your diagnosis, treatment options, and its impact on your quality of life that you didn't expect to happen?<br>• Did you do make any lifestyle changes (e.g., physical activity, healthy eating, reduce or quit smoking/drinking) before treatment? Why?	 *Environmental context and resources; Social influences*<br><br>*Knowledge*<br><br>*Beliefs about consequences; Intentions; Goals; Behavioural regulation*
Transition: Before the interview, we talked a little about 'cancer prehabilitation' ***which are steps that people can take to improve their health and wellbeing before receiving cancer treatment in hopes to reduce the side effects and complications of the treatment'*** This can include:<br><br>- Education on cancer treatment and recovery<br>- Exercise/physical activity<br>- Smoking and alcohol cessation<br>- Healthy eating/nutrition<br>- Mental health		

Perceived Interest in Cancer Prehabilitation	**Would you be interested in attending a program like this?**<br>• Do you think a prehabilitation program would benefit you?<br>• Of the components I listed, which ones do you think you would find most beneficial and least beneficial to you? Why?	*Beliefs about consequences; Intentions; Goals; Optimism*
Ideal Design and Delivery of Prehabilitation	**What would make you more like to join a prehabilitation program?**<br>• Do you feel confident that you could take part in prehabilitation activities?<br>• What would you need to know to want to participate in prehabilitation?<br>• Who would you like to deliver or how would you like to receive that information?<br>• What type of programming would interest you? E.g. walking group, yoga class, personal trainer, etc. Why?<br>• Would you prefer to have prehabilitation in-person or virtual? In a group setting or one-on-one?<br>• What are some benefits that would make you want to get involved in prehabilitation? E.g., having a support system, feeling better, reducing side effects<br>• Were there other things that would have got in the way of you being involved in prehabilitation?<br>• What do you think could be barriers to attending cancer prehabilitation? E.g., location, cost, time of day. What would make it easy?	*Skills; Beliefs about capabilities*<br>*Knowledge; Beliefs about Consequences*<br>*Social influences; Environmental context and resources*<br>*Environmental context and resources; Reinforcement; Emotion*<br>*Social influences; Environmental context and resources*<br>*Reinforcement; Social influences; Goals*<br>*Memory, attention and decision processes; Social/professional role*<br>*Environmental context and resources*
